# High HPV-51 Prevalence in Invasive Cervical Cancers: Results of a Pre-Immunization Survey in North Sardinia, Italy

**DOI:** 10.1371/journal.pone.0063395

**Published:** 2013-05-22

**Authors:** Andrea Piana, Giovanni Sotgiu, Clementina Cocuzza, Rosario Musumeci, Vincenzo Marras, Stefania Pischedda, Silvia Deidda, Elena Muresu, Paolo Castiglia

**Affiliations:** 1 Department of Biomedical Sciences, Hygiene and Preventive Medicine Unit, University of Sassari – AOU Sassari, Sassari, Italy; 2 Department of Surgery and Interdisciplinary Medicine, University of Milano Bicocca, Milan, Italy; 3 Department of Clinical and Experimental Medicine, University of Sassari, Sassari, Italy; Vanderbilt University, United States of America

## Abstract

**Background:**

Human Papilloma virus (HPV) is recognized as the etiological agent of benign and malignant ano-genital lesions. The most prevalent genotypes associated with cervical carcinoma are HPV-16 and -18 worldwide. However, recent studies have emphasized the role of other genotypes, such as HPV-51, in the pathogenesis of cervical dysplasia. The aim of the study was to estimate the burden of HPV-51 infection in invasive cervical malignant lesions in Northern Sardinia, Italy.

**Methods/Principal Findings:**

An observational, retrospective, prevalence, mono-center study was carried out to evaluate the presence of HPV genotypes in tissues biopsies of cervical lesions (CIN-1, CIN-2, CIN-3 and invasive carcinoma) gathered from 1996 to 2009. Biological samples were collected from women admitted consecutively to a tertiary university hospital situated in Sassari, Italy. Molecular methods were used to identify 28 oncogenic HPV types. A total of 155 formalin-fixed and paraffin-embedded cervical tissue samples were analyzed. Approximately half of the cervical lesions were classified as invasive carcinoma. HPV-DNA was detected in 71% of the samples, with a higher frequency (100%) in those categorized as invasive neoplasia. Mono- or co-infections were demonstrated in 45.8% and 25.8% of the cervical samples, respectively. Overall, the most prevalent HPV types were -16 (49%) and -51 (19.4%), with an increased frequency of detection associated with the severity of the cervical lesions.

**Conclusions/Significance:**

This survey highlights for the first time the relevant role of HPV-51 infection in the pathogenesis of invasive cervical cancer prior to the introduction of a vaccination program. Although a selection bias could have influenced the results, other recent studies have described the impact of HPV-51. This remarkable epidemiological element should be carefully evaluated, particularly in the view of opting for preventive vaccines, whose cross-protection patterns determine their efficacy in protecting against infection from HPV types that are not included in the vaccine itself.

## Introduction

Cervical cancer is deemed the third most frequent neoplasia in women worldwide; in particular, it is the second most prevalent cancer among women aged 15–44 years. Recently, it was estimated an annual number of 529,828 new cases of cervical carcinoma and of 275,128 deaths from cervical carcinoma [1].

Human Papilloma viruses (HPVs) have been shown to be associated with cervical cancers since 1976 [2], as a consequence of a persistent infection which occurs in less than 10% of the infected women [3]. The majority of sexually active people (∼75%) is infected by HPV types at some point during their lifetime course [4], [5]. The overall estimated female prevalence of HPV infection is 11.4% [1]. More than 70% of global cervical neoplasias have been described to be associated with HPV genotypes -16 and -18 [1].

On this basis, two prophylactic vaccines containing type -16 and -18 specific antigens were recently developed; to date, they have showed a 7–9 year clinical efficacy of about 100% against cervical cytological abnormalities caused by HPV-16 and -18 genotypes, with a variable cross-protective efficacy against cervical lesions associated with other high-risk oncogenic types [6], [7].

A prevalence study showed that other high-risk HPV genotypes than -16 and -18 (*i.e.*, HPV types -45, -33, -31, -52, -35, -39, -58, -51, -59, -56, and -66) were associated with more than 20% of cervical carcinomas globally [8], [9].

The epidemiology of circulating HPV genotypes was proved to be heterogeneous and the impact of the vaccines could vary depending on the regional distribution of HPV types [8].

Recently, our group performed a cross-sectional investigation, which described a high overall prevalence of HPV genotype -16 (20.5%) and -51 (14.3%) in Northern Sardinia, Italy. Their presence, as single or coinfecting agents, was detected in all kind of cytological lesions; in particular, HPV types -16 and -51 DNA were detected in 12.5 and 6.3% of atypical sqamous cells of undetermined significance; 24.0% and 16.0% of low-grade squamous intraepithelial lesions; 50.0% and 50.0% of high-grade squamous intraepithelial lesions, respectively [8]. A similar epidemiological pattern was also described by other previous studies [11], [12], [13], [14].

Since an unexpected proportion of some genotypes could be due to random variability in limited geographical areas, in order to evaluate the epidemiological dynamics of HPV-51, particularly in invasive cervical lesions, we investigated the presence of HPV DNA in embedded tissues samples of cervical lesions collected in the same area of Northern Sardinia, Italy, before and during the above-mentioned prevalence study.

## Methods

### Ethics Statement

Ethical approval and informed consent for this study was unnecessary, according to the Italian legislation concerning the guidelines for the performance of observational studies (G.U. n. 76. 31-3-2008). However, a formal approval of the study protocol was requested in 2007 to the Ethical Committee of the Azienda Sanitaria Locale n°1 of Sassari, Italy (PN-132, 2007).

### Design of the Study

An observational, retrospective, cross-sectional, mono-center study was performed in Northern Sardinia, Italy, to evaluate the prevalence of HPV infection in a subset of embedded cervical lesions (Cervical Intra-epithelial Neoplasia – CIN-1, 2, 3, and invasive carcinoma), consecutively collected from 1996 to 2009 and belonging to women admitted to a tertiary university hospital located in Sassari, Italy.

An *ad-hoc* case report form was created to record hysto-pathological and virological information. Data were anonymized in compliance with the Law Decree No. 196/2003, article 24 (Code for the protection of personal data).

### Sample Collection and Virological Analysis

HPV-51 infection was expected to account for 1% of invasive cervical cancers, on the basis of the estimates of a recent international prevalence study [8]. Considering the high prevalence detected in our previous study (*i.e.*, 50% in high-grade squamous intraepithelial lesions), an estimated number of three invasive carcinomas per year was needed to obtain a statistically significant difference. Therefore, a sample of 69 formalin-fixed and paraffin-embedded tissues from invasive cervical carcinoma biopsies, collected from 1996 to 2009, was selected. Concomitantly, it was decided to confirm results obtained in our previous prevalence study [10] by including tissue-embedded samples from cervical lesions other than invasive neoplasias (∼30 samples for each grade of cervical histological lesion: CIN-1, CIN-2 and CIN-3), as well as to exclude possible selection bias between populations with and without invasive carcinoma.

DNA was isolated after extraction and purification using the DNeasy kit (Qiagen, Valencia, CA) [15]. Every section was heated for 20 minutes at 120°C in 180 µl ATL lysis buffer and, then, incubated at 65°C with Proteinase K overnight. DNA was eluted in 100 µL and analyzed instantaneously or after a variable storage at −20°C. [15].

DNA was tested using INNO-LiPA HPV Genotyping Extra, specific for 28 HPV genotypes (-6, -11, -16, -18, -26, -31, -33, -35, -39, -40, -43, -44, -45, -51, -52, -53, -54, -56, -58, -59, -66, -68, -69, -70, -71, -73, -74, -82) [10].

When positivity for HR genotypes -16, -18, -31, -45, -51, and -52 was found, confirmation was obtained testing samples with an "in-house" Real-Time quantitative TaqMan PCR assay [10], [16].

Cross-protection against cervical carcinoma from preventive vaccines was estimated using statistically significant findings showed in the studies PATRICIA and FUTURE II for the bivalent and tetravalent vaccines, respectively [17], [18].

### Statistical Analysis

Qualitative variables were summarized with percentages and statistically compared using the z-proportion hypothesis test. Linear trend for proportions was adopted to test the pattern of HPV genotypes in different cervical lesions. 95% confidence intervals were calculated to estimate HPV genotype prevalence.

Logistic regression analysis was performed to assess the role of potential variables, including infections by several HPV genotypes, in the development of invasive cervical carcinoma.

A p-value <0.05 was considered statistically significant.

All the analyses were carried out using the statistical software Stata 11.0 (StataCorp LP, College Station, Texas).

## Results

Cervical tissue samples, obtained during the time period 1996–2009, from 155 women, whose mean (standard deviation) age was 43.71 (14.68) years, were included in the study.

The majority of the women were affected by invasive carcinoma (69, 44.5%), followed by CIN-1 (30, 19.4%), CIN-2 (29, 18.7%), and CIN-3 (27, 17.4%).

A total of 110 out of 155 (71%) embedded-tissues were found to be positive for HPV-DNA, ranging from 6 (20%) in CIN-1 to 69 (100%) in invasive cervical neoplasia (p-value for linear trend <0.001) (Table 1).

**Table 1:** Prevalence of high- and low-risk oncogenic HPV types stratified by histological results.

		HPV 16		HPV 18		HPV 51		Other than HPV 16 and 51 HR types		Nearest non-vaccine types (31, 33, 45, 52, 58)		HR types		LR types		Any HPV type		Mono-infection		Co-infection with HR and/or LR types	
		*n (%)*	*95% CIs*	*n (%)*	*95% CIs*	*n (%)*	*95% CIs*	*n (%)*	*95% CIs*	*n (%)*	*95% CIs*	*n (%)*	*95% CIs*	*n (%)*	*95% CIs*	*n (%)*	*95% CIs*	*n (%)*	*95% CIs*	*n (%)*	*95% CIs*
*Histology*	*CIN1*	4 (13.3)	1.1–25.4	0 (0.0)	0.0–0.0	0 (0.0)	0.0–0.0	3 (10.0)	−0.7–20.7	2 (6.7)	−2.3–15.6	6 (20.0)	5.7–34.3	0 (0.0)	0.0–0.0	6 (20.0)	5.7–34.3	5 (16.7)	3.3–30.0	1 (3.3)	−3.1–9.8
	*CIN2*	2 (6.9)	−2.3–16.1	0 (0.0)	0.0–0.0	3 (10.3)	−0.7–21.5	7 (24.1)	8.6–39.7	7 (24.1)	8.5–39.7	10 (34.5)	17.2–51.8	0 (0.0)	0.0–0.0	10 (34.5)	17.2–51.8	8 (27.6)	11.3–43.9	2 (6.9)	−2.3–16.1
	*CIN3*	17 (63.0)	44.7–81.1	3 (11.1)	−0.7–22.7	2 (7.4)	−2.5–17.3	11 (40.7)	22.2–59.3	8 (29.6)	12.4–46.8	25 (92.6)	82.7–100.0	1 (3.7)	−3.4–10.8	25 (92.6)	82.7–100.0	18 (66.7)	48.9–84.5	7 (25.9)	9.4–42.5
	*Invasive carcinoma*	53 (76.8)	66.9–86.8	3 (4.4)	−0.5–9.2	25 (36.2)	24.9–47.6	20 (29.0)	18.3–39.7	15 (21.7)	0.8–42.6	69 (100.0)	100.0–100.0	8 (11.6)	4.0–19.1	69 (100.0)	100.0–100.0	40 (58.0)	46.3–69.6	29 (42.0)	30.4–53.7
*All screened sample*		76 (49.0)	41.2–56.9	6 (3.9)	0.8–6.9	30 (19.4)	13.1–25.6	41 (26.5)	19.5–33.3	32 (20.6)	14.2–27.0	110 (71.0)	63.8–78.1	9 (5.8)	2.1–9.5	110 (71.0)	110 (71.0)	71 (45.8)	38.0–53.7	39 (25.2)	18.4–32.0
*p-value for trend*		<0.0001		0.10		<0.0001		0.06		0.16		<0.0001		0.04		<0.0001		<0.0001		<0.0001	

CIs: Confidence Intervals.

CIN: Cervical Intra-Epithelial Neoplasia.

HR: High-Risk genotype.

LR: Low-Risk genotype.

High-risk genotypes were detected in all HPV-DNA positive samples (110, 100%), whereas low-risk genotypes were detected in only 9 cases (9, 5.8%). A higher percentage of tissue positivity for high-risk HPV oncogenic types was demonstrated in CIN-3 lesions and invasive neoplasias (92.6% and 100%, respectively).

The prevalence of HPV infection significantly increased alongside with the age: 25% (2/8) in those aged 15–24 years, 52.5% (21/40) in those aged 25–34 years, 68.3% (28/41) in those aged 35–44 years, 82.9% (29/35) in those aged 45–54 years, 93.3% (14/15) in those aged 55–64 years, 100.0% (10/10) in those aged 65–74 years, 100.0% (6/6) in those aged over 75 years (slope 0.13; p-value for linear trend <0.0001). An indistinguishable proportional linear trend was observed for the prevalent high-risk genotypes.

Infection by a single HPV genotype was described in about half of the samples (71, 45.8%); co-infection with multiple high-risk and/or low-risk genotypes was found in 40 (25.8%) of cervical samples, with an increasing linear trend associated with the severity of the histo-pathological pattern (p-value for linear trend <0.001).

The most prevalent genotypes were HPV-16 (76, 49.0%) and HPV-51 (30, 19.4%), which accounted for 64.6% of all detected genotypes. HPV-16 and/or -51 infection was proved in 75.5% (83/110) of all HPV-DNA positive samples. The positivity significantly increased alongside with the severity of the cervical lesions for the genotypes HPV-16 and -51 (p-value for linear trend <0.001). Other relevant HPV genotypes were: 45 (52, 5.8%), 31 (7, 4.5%), 69 (7, 4.5%), 71 (7, 4.5%), and 18 (6, 3.9%). The observed prevalence of HPV-16/51 co-infection (23/155, 14.8%) was almost similar to that estimated on the basis of single HPV-16 and HPV-51 infections (14.7%; p-value >0.05). Only a few patients were co-infected by HPV genotypes -16/18 (4, 2.6%).

Among 71 patients infected by a single genotype, the most frequent were: HPV-16 (41, 57.8%), -51 (5, 7.0%), -31 (4, 5.6%), -45 (4, 5.6%), and -18 (2, 2.8%).

Cervical carcinomas were positive for HPV-16 and -51 in 76.8% and 36.2%, respectively; the proportion of co-infected cases was 20.9%. Therefore, the percentage of cervical carcinomas with at least HPV-16 and/or 51 infection was 81.2% (56/69). The estimated proportion of cases potentially protected by the current vaccines (*i.e.*, HPV-16, -18, -31, -33, -45, and -51) is 92.8%.

In particular, cross-protection against HPV-16 and -18 nearest genotypes (*i.e.*, HPV-31, -33, -45, -51), conferred by the current preventive bivalent and tetravalent vaccines, could be 11.4% and 1.5%, respectively, based on cross-protective efficacy data [17], [18].

A stepwise logistic regression analysis was carried out in order to assess the potential covariates associated with the occurrence of invasive cervical carcinoma (Table 2). The univariate analysis showed a statistically significant role of the HPV genotypes -16 (OR: 9.07; p-value <0.0001), -51 (OR: 9.20; p-value <0.0001), and -45 (OR: 11.14; p-value: 0.025); the adjusted analysis confirmed the significant association for the HPV genotypes -16 (OR: 19.82; p-value <0.0001), -51 (OR: 11.32; p-value: 0.004), and -45 (OR: 22.07; p-value: 0.033).

**Table 2:** Logistic regression analysis of the covariates associated with the invasive cervical carcinoma.

HPV genotype	Univariate analysis		Adjusted* analysis	
	*OR*	*p-value (95% CIs)*	*OR*	*p-value (95% CIs)*
*HPV-16*	9.07	<0.0001 (4.34–18.92)	19.82	<0.0001 (5.55–70.82)
*HPV-18*	1.26	0.78 (0.25–6.44)	–	–
*HPV-33*	1.91	0.49 (0.31–11.76)	–	–
*HPV-45*	11.14	0.025 (1.35–91.4)	22.07	0.033 (1.29–378.83)
*HPV-51*	9.20	<0.0001 (3.29–25.72)	11.32	0.004 (2.13–60.17)
*Increasing age, years*	1.16	<0.0001 (1.11–1.22)	1.19	<0.0001 (1.12–1.27)

* Adjusted for the main confounding variables such as increasing age.

OR: Odds Ratio.

CIs: Confidence Intervals.

**Figure 1:**
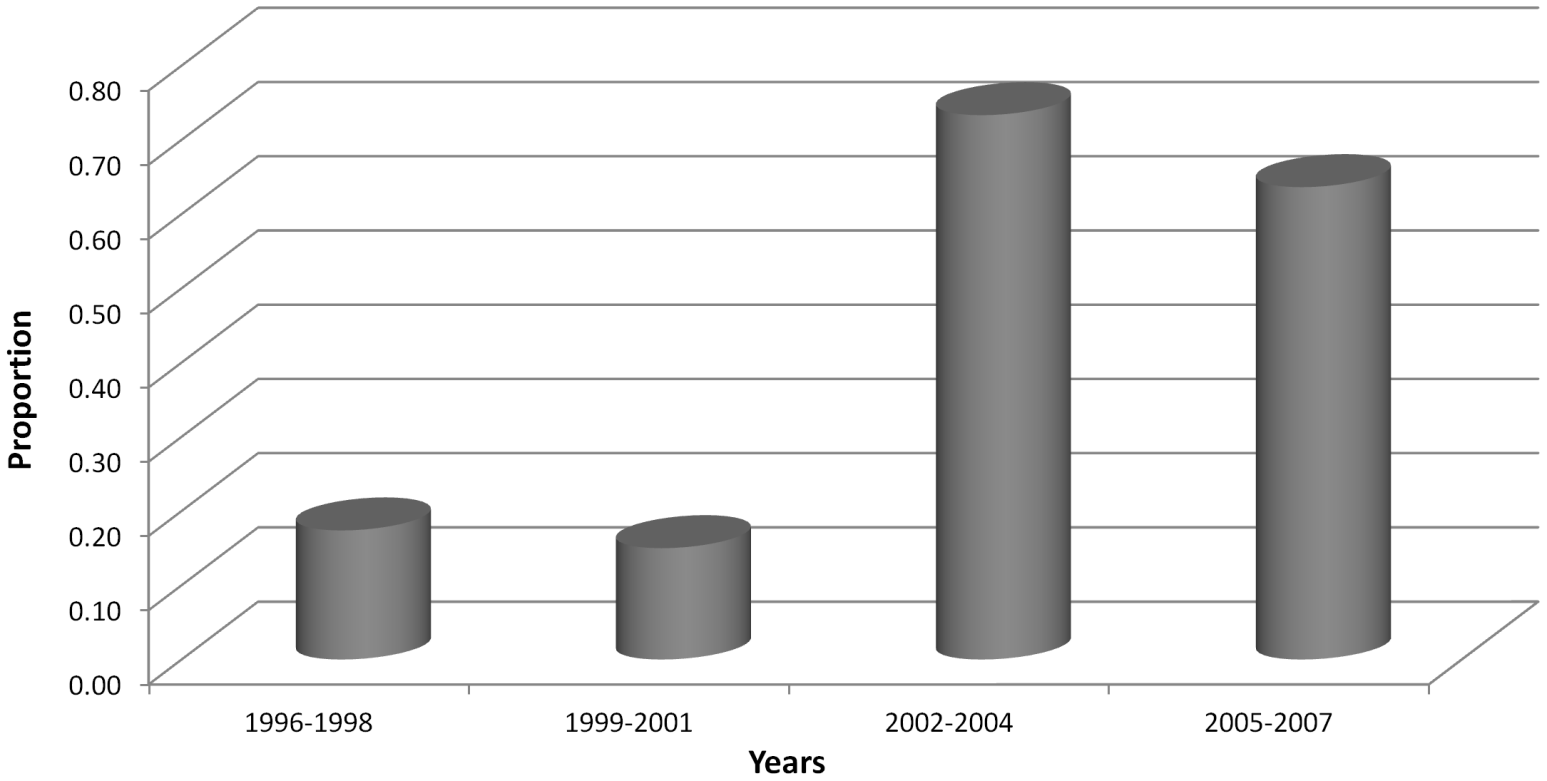
Relative frequency of the HPV-51 genotype in invasive cervical carcinomas from 1996 to 2007.

The proportional distribution of the main HPV genotypes (*i.e.*, -16, -51, and -45) responsible of invasive cervical carcinoma did not significantly change during the study period (p-values for linear trend >0.05); however, the analysis of the temporal trend of the only HPV-51 genotype prevalence, in mono- or co-infection, significantly changed due to non-random variability (chi-square for trend of proportions; p-value <0.0002) (Figure 1). The retrospective nature of the study did not allow further analyses to assess the role of covariates in the proportional variability of the main genotypes.

## Discussion

The present study confirms the primary role of HPV-16 in the pathogenesis of cervical carcinoma and describes an unusual high prevalence of HPV-51 infection (36.2%) in embedded invasive cervical neoplasias in Northern Sardinia, Italy, in a pre-vaccination period (1996–2007). It confirms that HPV-51, rarely detected in other geographical areas [8], [19], is a frequent cause of cervical lesions in Northern Sardinia, as previously showed [10]. Furthermore, the present survey indicates that HPV-51 prevalence increases alongside with the cervical lesion severity (from 0% in CIN-1 lesions to 36.2% in invasive carcinomas; p-value <0.05) as well as increasing age significantly correlates with lesion severity, explaining the increased HPV-51 prevalence trend alongside with increasing age in our setting.

To our knowledge, this is the first study pointing out such a strong association between HPV-51 and invasive cervical cancer (OR 9.2; IC95%: 3.3–25.7).

As previously discussed [10], three major hypotheses could be provided to clarify the estimated relevant epidemiological impact of HPV-51 in our territory: a) a possible selection bias due to a voluntary based PAP test screening instead of a systematic one, that might have enrolled patients with an higher prevalence of symptoms; b) a random temporal variability in this restricted geographical area; c) a true higher prevalence of HPV-51 infection and/or, conversely, an under-reporting of its prevalence in other areas.

With regards to a potential selection bias, it was already showed that, even if this possibility cannot be excluded, it may not play a role, as no significant differences were detected between HPV positive and negative women in terms of PAP test compliance in the years preceding our survey [10].

On the other hand, if the high prevalence demonstrated for HPV-51 is due to a random variability in this restricted geographic area, it should have been lower in the past, in particular in cervical neoplasias occurring several decades after the HPV infection. Therefore, the high prevalence of HPV-51 in embedded tissues from invasive cervical carcinomas, with a significant temporal differences since 1996 up to recent years, strongly indicates that this HPV genotype has been endemic in Northern Sardinia at least for the last thirty years.

On this basis, the high HPV-51 prevalence could be deemed a peculiar epidemiological feature of Northern Sardinia. However, the higher diagnostic accuracy of the molecular methods implemented in our study might have positively affected the sensitivity of the survey, explaining, consequently, the differences found comparing our findings with the results of other studies, which used different diagnostic techniques [19]. Recent epidemiological studies showing high HPV-51 frequency in several geographical areas could support the hypothesis of previous underreporting for some HPV types owing to less sensitive tools [20], [21], [22], [23], [24], [25], even if HPV-51 is frequently detected in co-infection with other types mainly in developing countries, characterized by a higher HPV genotype variability and HIV-infection [26], [27], [28], [29], [30]. Based on this assumption, the epidemiological differences identified in several areas might be reduced in the near future scaling-up new sensitive methods.

Results from this epidemiological study indirectly indicate that current vaccines may prevent infection by HPV genotypes circulating in Northern Sardinia, particularly as a result of the use of the bivalent vaccine owing to its larger cross-protective effect against HPV genotypes, including HPV-51. Current available vaccines are in fact believed to prevent more than 70% of the cases of cervical carcinoma owing to their cross-protection activity. Nevertheless, it is presently unclear if data on cross-protective efficacy, obtained from clinical trials, indicates long-lasting immunity [31]. Clinical trials on HPV vaccines were primarily designed to test the efficacy of immunization against HPV genotypes -16 and -18. The evaluation of cross-protection was only a secondary objective; consequently, the sample size was computed to statistically evaluate the primary objective. However, it is relevant to highlight the overall elevated efficacy of HPV vaccines against CIN2 and CIN3 lesions, irrespective of HPV genotypes.

However, the introduction of HPV vaccines in the Italian market, followed by the implementation of a specific public health program, could modify the epidemiological scenario, favoring the spread of HPV genotypes whose antigens are not included in the vaccines. This has been described for other bacterial infections following the introduction of vaccination programmes [32], even if the replacement phenomenon sporadically described for HPV [33] is considered to have a very low probability of occurrence [34].

Data obtained from this prevalence survey will allow to evaluate the possible epidemiological impact of HPV vaccine on the etiological dynamics of cervical cancer; in particular, to determine whether the implementation of a vaccination program based on the bivalent (HPV-16, HPV-18) vaccine in Northern Sardinia can reduce the burden of HPV-51 infection as a consequence of its cross protective efficacy against HPV-31, -33, -45, and -51 [7].

Several limitations can be indentified in the survey carried out in Northern Sardinia: firstly, the cross-sectional nature of this study could not permit to adequately evaluate the dynamics of the HPV genotypes circulation in North Sardinia, being an observational, prospective design more suitable to recognize temporal changes. Nevertheless, the 10-year period selected for this study is deemed to be sufficient to give an overview of the HPV genotype prevalence in the pre-vaccination era, when, theoretically, no selective pressure for ecological changes occurred in this geographical area.

The confidence intervals of the estimates of the prevalence data show a wide range for some of the less frequent HPV genotypes, underlying an issue of poor statistical power; therefore, an under-reporting of cervical lesions, particularly CIN lesions, could be hypothesized, although samples of all invasive cervical carcinomas, notified in the study period to the official Northern Sardinian registry, were collected to undergo molecular analysis for HPV-DNA. Moreover, we consecutively enrolled samples of individuals complaining of clinical symptoms and, consequently, a selection bias could not be excluded, though our previous prevalence study [10] did not identify any statistical difference between women with and without symptoms in terms of compliance to PAP smear testing.

In conclusion, as previously reported, this study has confirmed the high prevalence of HPV infection in women with cervical dysplasia and invasive disease in our region; moreover, it highlighted the role of HPV-51 in determining invasive disease, emphasizing the importance of orienting immunoprophylaxis towards this HPV type in the prevention of cervical cancer in our geographical area.
